# Comparison of humoral and cellular immune responses between ChAd-BNT heterologous vaccination and BNT-BNT homologous vaccination following the third BNT dose: A prospective cohort study

**DOI:** 10.3389/fimmu.2023.1120556

**Published:** 2023-03-02

**Authors:** Wooho Sim, Hyunhye Kang, Jin Jung, Jihyun Lee, Geon Young Ko, Hye-Sun Park, Jeewan Choi, Kinam Park, Eun-Jee Oh

**Affiliations:** ^1^ Department of Internal Medicines, The Armed Forces Capital Hospital, Seongnam, Republic of Korea; ^2^ Department of Laboratory Medicine, Seoul St. Mary’s Hospital, College of Medicine, The Catholic University of Korea, Seoul, Republic of Korea; ^3^ Resesarch and Development Institute for In Vitro Diagnostic Medical Devices, College of Medicine, The Catholic University of Korea, Seoul, Republic of Korea; ^4^ Department of Biomedicine & Health Sciences, Graduate School, The Catholic University of Korea, Seoul, Republic of Korea; ^5^ Infectious Disease Response Division, Armed Forces Medical Command, Seongnam, Republic of Korea; ^6^ Medical Corps, Republic of Korea Army, Gapyeong, Republic of Korea

**Keywords:** SARS-CoV-2, vaccine, heterologous, immune response, breakthrough infection

## Abstract

**Introduction:**

The differential immune responses after two additional BNT162b2 (BNT) booster doses between ChAdOx1 nCoV-10 (ChAd)-primed and BNT-primed groups have not been elucidated. The aim of this study was to compare vaccine-induced humoral and cellular immune responses and evaluate breakthrough infection between the two vaccination strategies.

**Methods:**

In 221 healthy subjects (111 in the ChAd group), longitudinal immune responses were monitored at 3, 4, and 6 months after the 2nd dose and 1, 3, and 6 months after the 3rd dose. Humoral immunity was measured by two fully automated chemiluminescent immunoassays (Elecsys and Abbott) and a surrogate virus neutralization test (sVNT). Cellular immunity was assessed by two interferon-γ (IFN-γ) release assays (QuantiFERON SARS-CoV-2 and Covi-FERON).

**Results:**

After the 2nd dose of BNT vaccination, total antibody levels were higher in the ChAd group, but IgG antibody and sVNT results were higher in the BNT group. Following the 3rd dose vaccination, binding antibody titers were significantly elevated in both groups (ChAD-BNT; 15.4 to 17.8-fold, BNT-BNT; 22.2 to 24.6-fold), and the neutralizing capacity was increased by 1.3-fold in both cohorts. The ChAd-BNT group had lower omicron neutralization positivity than the BNT-BNT group (P = 0.001) at 6 months after the 3rd dose. Cellular responses to the spike antigen also showed 1.7 to 3.0-fold increases after the 3rd dose, which gradually declined to the levels equivalent to before the 3rd vaccination. The ChAd cohort tended to have higher IFN-γ level than the BNT cohort for 3-6 months after the 2nd and 3rd doses. The frequency of breakthrough infection was higher in the ChAd group (44.8%) than in the BNT group (28.1%) (P = 0.0219). Breakthrough infection induced increased humoral responses in both groups, and increase of cellular response was significant in the ChAd group.

**Discussion:**

Our study showed differential humoral and cellular immune responses between ChAd-BNT-BNT heterologous and BNT-BNT-BNT homologous vaccination cohorts. The occurrence of low antibody levels in the ChAd-primed cohort in the humoral immune response may be associated with an increased incidence of breakthrough infections. Further studies are needed on the benefits of enhanced cellular immunity in ChAd-primed cohorts.

## 1 Introduction

Vaccination against severe acute respiratory syndrome coronavirus 2 (SARS-CoV-2) has been the key strategy to protect against coronavirus disease 2019 (COVID-19), and 11 vaccines have been approved for emergency use through the World Health Organization (WHO) (accessed September 2022) (https://covid19.trackvaccines.org/agency/who/). Available and globally distributed vaccines are based on different platforms (1), and heterologous vaccination schedules are not uncommon (2, 3). Among the authorized vaccines, an adenoviral vector-based vaccine (ChAdOx1 nCoV-10, AstraZeneca; hereafter referred to as ChAd) and an mRNA vaccine (BNT162b2, Pfizer-BioNTech; hereafter referred to as BNT) have been approved as a two-dose homologous primary series in many countries. Yet, the general concerns on adverse thromboembolic events with ChAd (4, 5) prompted a subsequent need to change the booster vaccination platform, particularly in young adults, and the immune responses following heterologous vaccination in ChAd-primed groups need to be addressed (6, 7). In Korea, four vaccines have been approved for use, with BNT and ChAd being the most widely used. Previous studies reported that a heterologous schedule with ChAd-BNT elicited a higher immune response compared to ChAd-ChAd (2, 8). The initial immune responses declined over time, and the pandemic spread of immune-evasive variants of concern (VOC), including omicron, required a 3^rd^ dose worldwide. A German study reported that the decreasing immune responses were effectively restored after a 3^rd^ BNT vaccination in previously ChAd-BNT-vaccinated individuals (9). However, few longitudinal studies have been conducted on the differential humoral and cellular immune responses after the 3^rd^ BNT administration in a ChAd-BNT heterologous primary schedule compared to the BNT-BNT primary series. In addition, the differences in incidence of breakthrough infections in these two groups and the immune kinetics of individuals with breakthrough infections have not yet been fully elucidated.

In this study, we compared the humoral and cellular immune responses between ChAd-BNT-BNT and BNT-BNT-BNT vaccination groups using blood samples sequentially collected from 3 months after the 2^nd^ dose to 6 months after the 3^rd^ BNT vaccination. For a subset of participants with breakthrough infection, we separately analyzed immune responses following infection, and neutralization activity against omicron was compared with that of infection-naïve participants.

## 2 Material and methods

### 2.1 Human subjects

**Figure 1:**
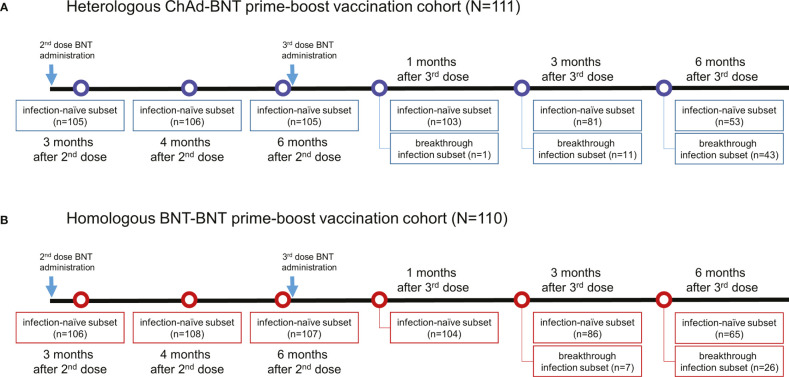
Overall timeline of sampling and the number of participants in ChAd-primed **(A)** and BNT-primed **(B)** vaccination cohorts. N, total number of participants in each cohort; n, number of participants who underwent blood sampling during the specified timepoint.

This study included 221 subjects who had received 2 doses of vaccination (111 heterologous ChAd-BNT- and 110 homologous BNT-BNT-vaccinated individuals) by July 2021 without previous SARS-CoV-2 infection. Prior SARS-CoV-2 infection was excluded by clinical history and negative SARS-CoV-2 nucleocapsid antibody in collected samples. They received a 3^rd^ dose of BNT vaccination 6 months after the 2^nd^ dose and were monitored until 6 months after the 3^rd^ dose. Blood samples were collected 6 times between September 2021 and June 2022 at an army division. Humoral and cellular immunity tests were performed at Seoul St. Mary’s Hospital. The sampling time, sampling interval, and number of samples are detailed in **Figure 1**. All participants were healthy volunteers working for the military service at a same army division, of which 16 participants reported well-controlled comorbidities. Participants were classified based on primary vaccine regimen, and baseline characteristics are summarized in **Table 1**. A subset of participants who experienced breakthrough infection during the study period was analyzed separately. Breakthrough infection was defined based on confirmed COVID-19 positivity or SARS-CoV-2 nucleocapsid antigen-specific antibody positivity. The COVID-19 confirmatory tests included reverse transcription polymerase chain reaction (RT-PCR) or rapid antigen test by an expert in accordance with the COVID-19 diagnostic guidelines of the Korean Society for Laboratory Medicine and the Korea Centers for Disease Control and Prevention (10). All participants provided written informed consent to use of their samples for research purposes. The study was approved by the Institutional Review Board of the Armed Forces Medical Command (AFMC-202202-BR-012-01).

**Table 1:** Baseline characteristics of study cohorts classified by vaccine schedule.

	Heterologous ChAd-BNT* vaccination cohort (n=111)	Homologous BNT-BNT* vaccination cohort (n=110)
Age, years		
Mean ± SD	39.4 ± 6.6	25.9 ± 2.4
Median (range)	40.0 (30.0 - 53.0)	25.5 (20.0 - 30.0)
Sex, n (%)		
Male	106 (93.8)	91 (82.7)
Female	7 (6.2)	19 (17.3)
Comorbidities†, n		
Chronic hepatitis B carrier	1	0
Diabetes	2	0
Hypertension	13	0
3^rd^ dose BNT vaccination, n (%)	108 (97.3)	105 (95.5)
Vaccination schedule		
First to second dose interval (days)		
Median (range)	78 (57-84)	21 (21-24)
Second to third interval (days)		
Median (range)	162 (151-178)	169 (165-182)
Breakthrough infection, n (%‡)	43 (44.8)	26 (28.1)
Symptomatic infection with positive confirmatory tests§		
	36 (83.7)	23 (88.5)
Asymptomatic infection with positive SARS-CoV-2 nucleocapsid antibody assay∥		
	7 (16.3)	3 (11.5)
Time of breakthrough infection, n		
Within 1 month posterior to 3^rd^ dose administration		
	1	0
Between 1 - 3 month – window posterior to 3^rd^ dose administration		
	10	7
Between 3 - 6 month – window posterior to 3^rd^ dose administration		
	32	19

*ChAd, ChAdOx1 nCoV-19 vaccine; BNT, BNT162b2 vaccine

† Comorbidity status was based on the relevant participants’ self-report, and all reported to be well-controlled.

‡ The percentages were calculated based on the modified per-protocol population.

§ The confirmatory tests include reverse transcription polymerase chain reaction (RT-PCR) or rapid antigen test by healthcare providers at authorized medical centers. Either diagnostic procedures were conducted according to Korean Society for Laboratory Medicine and the Korea Disease Prevention and Control Agency guidelines for diagnosing COVID-19.

**∥**Anti-SARS-CoV-2 nucleocapsid (Roche Diagnostics, Basel, Switzerland) assay was performed.

### 2.2 SARS-CoV-2 binding antibody detection

SARS-CoV-2 binding antibodies were measured using two commercial chemiluminescent immunoassay (CLIA) kits. The Elecsys Anti-SARS-CoV-2 assay (Roche Diagnostics, Basel, Switzerland) measures the total antibodies, while the SARS-CoV-2 IgG assay (Abbott, Chicago, IL, USA) measures the level of IgG antibodies. All assays were performed according to the manufacturer’s instructions. Binding antibodies were measured as units per mL (U/mL), and SARS-CoV-2 binding antibody units per mL (BAU/mL) were calculated according to the WHO international standard for anti-SARS-CoV-2 immunoglobulin using conversion factors (Roche 1.028, Abbott 0.142) (11–13). The cut-off value of 0.8 U/mL was applied for the Roche total antibody assay, while 50 AU/mL was provided as a cut-off for the Abbott IgG binding antibody assay. To define cases of SARS-CoV-2 breakthrough infection, we further performed a Roche assay detecting anti-SARS-CoV-2 nucleocapsid (NC) antibodies in samples obtained at 6 months after the 3^rd^ dose. If the sample was anti-NC antibody positive, we performed the same analysis using all samples previously collected from the same participant to determine the timing of breakthrough infection.

### 2.3 SARS-CoV-2 surrogate virus neutralization test

The SARS-CoV-2 surrogate virus neutralization test (sVNT) (GenScript, Piscataway, NJ, USA) was used to determine the % inhibition of neutralizing antibodies. The assay measures antibody-mediated blockage between the wild-type angiotensin-converting enzyme 2 (ACE2) receptor and SARS-CoV-2 receptor-binding domain (RBD) based on a competitive enzyme-linked immunosorbent assay (ELISA). The cut-off of ≥30% inhibition was applied according to the manufacturer’s instructions. For samples collected 6 months after the 3^rd^ vaccination, neutralization activity was further evaluated using sVNT targeting the SARS-CoV-2 omicron variant (GenScript) when the variant was rapidly spreading.

### 2.4 SARS-CoV-2 specific T-cell responses

Cellular immune responses were measured by SARS-CoV-2-specific interferon-γ (IFN-γ) release assay (IGRA) using two commercially available kits: Covi-FERON (SD Biosensor, Suwon, Korea) and QuantiFERON (QIAGEN, Germantown, MD, USA). Both IGRA kits require the use of special antigen-sensitized collection tubes. The Covi-FERON test contains five tubes: an original spike protein (SP) antigen tube (O-Sp), variant SP antigen tube (V-Sp), nucleocapsid protein antigen tube (NP), Nil tube, and mitogen tube. The original spike protein tube contains spike protein derived from Wuhan and UK variants (B.1.1.7), while the variant spike protein tube contains the spike protein from South Africa (B.1.351) and Brazil (P.1) strains (14). Mitogen tubes were used as positive controls, and the Nil tubes were used as negative background values. After incubating 1 mL of whole blood in each collection tube at 37˚C for 16 to 24 hours, the amount of IFN-γ (IU/ml) was measured in plasma samples by ELISA. The final IFN-γ values in each tube were calculated after subtracting the IFN-γ concentration in the Nil tubes. The cut-off value of 0.25 IU/mL was applied as previously described (14).

Regarding the QuantiFERON assay, blood samples were drawn into each set of QuatiFERON Control set blood collection tubes, consisting of two SARS-CoV-2 antigen-specific tubes, one Nil tube, and a Mitogen tube. The SARS-CoV-2-specific tubes consist of two SARS-CoV-2 spike peptide pools (Ag1 and Ag2) (15). The Ag1 tube contains CD4+ epitopes derived from the S1 unit of the S protein, and the Ag2 tube contains CD4+ and CD8+ epitopes from the S1 and S2 subunits. The amount of IFN-γ was measured by ELISA in the plasma from each tube, and the final reported value was determined after subtracting background Nil IFN-γ level. All assays were performed in accordance with the manufacturer’s instructions.

### 2.5 Statistical analysis

Continuous data were not normally distributed, presented as geometric mean with 95% confidence interval, compared by rank sign tests (Mann–Whitney U -test or Wilcoxon test). When calculating the geometric mean was not possible due to values of 0 or less, the arithmetic mean was used. Categorical data are presented as count and percentage, and data were compared with χ²-tests. Statistically significant changes over time within each group were estimated by repeated measure two-way analysis of variance (RM-ANOVA) with Geisser-Greenhouse correction for sphericity and *post hoc* analysis with Sidak’s test for multiple comparisons. Spearman rank correlation was used to compare quantitative values from different assays. Data analysis and visualization were performed using Prism version 9.4.1 for Windows (GraphPad, San Diego, CA, USA) or MedCalc statistical software version 20.114 (MedCalc Software Ltd, Ostend, Belgium), and *P* value <0.05 (two-tailed) was considered statistically significant.

## 3 Results

### 3.1 Humoral response in infection-naïve participants

**Figure 2:**
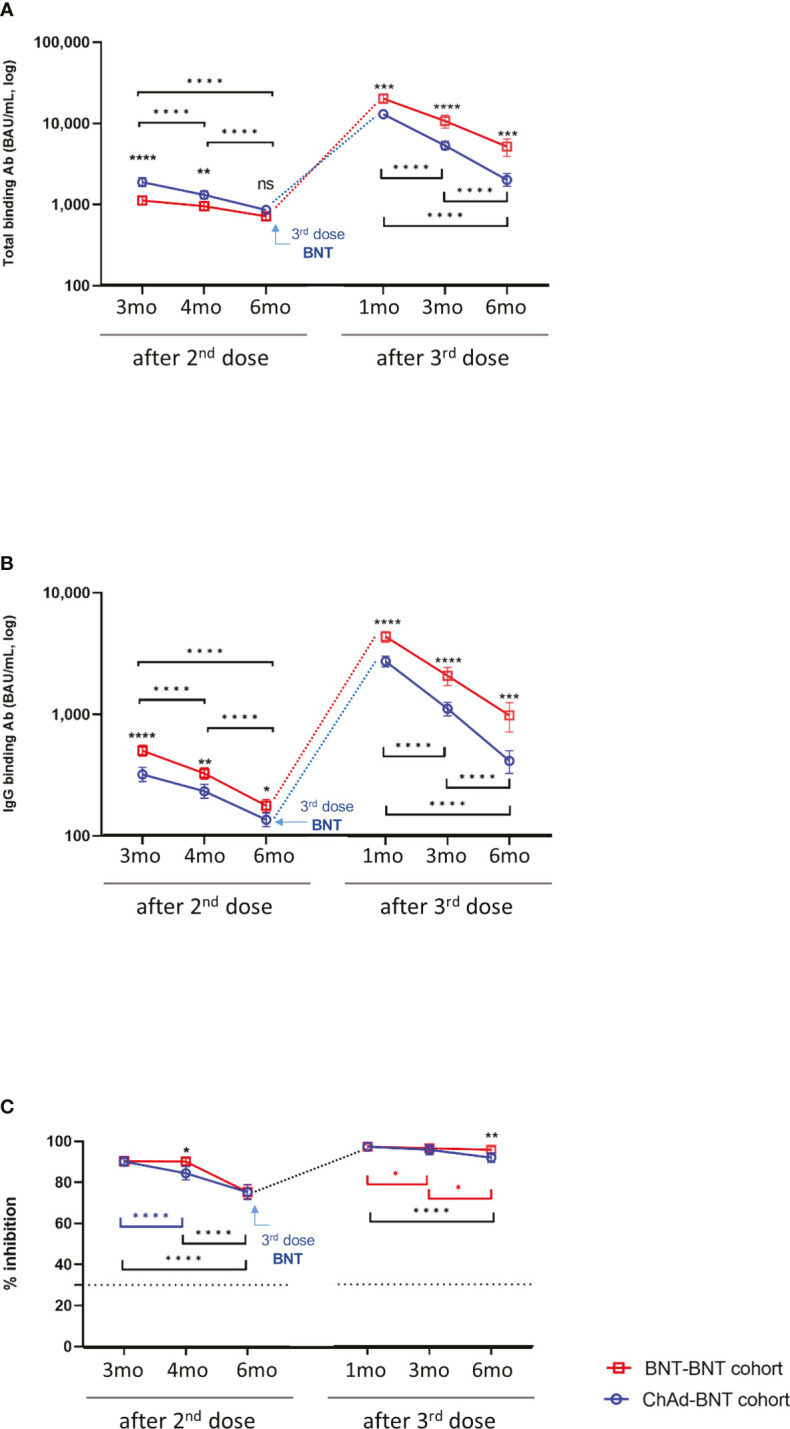
Binding antibody and neutralization responses in infection-naïve participants. SARS-CoV-2 spike-specific total binding antibody titer **(A)** and IgG binding antibody **(B)** and % inhibition by surrogate virus neutralization test **(C)** are shown. Data are presented as geometric mean with 95% confidence interval. The statistical significance was calculated using repeated measure two-way ANOVA with Sidak’s multiple comparisons test. When the ChAd-BNT cohort and BNT-BNT cohort revealed significant differences, the significance is expressed either over the blue circle or over the red square. When significant change was observed over a certain period, it is expressed using ticked lines (blue: Chad-BNT cohort, red: BNT-BNT cohort, black: both cohorts). The assay cut-off is presented as a horizontal dotted line. ns, not significant; *P<0.05; **P<0.01; ***P<0.001; ****P <0.0001.

All participants showed SARS-CoV-2-specific binding antibody positivity at all time points when the manufacturer-provided cut-offs were applied (**Figure 2**). The changes in humoral immune responses were analyzed after the 2^nd^ and 3^rd^ vaccination doses.

When the changes in humoral responses after the 2^nd^ dose were analyzed in each cohort, total SARS-COV-2 spike antigen-specific and IgG antibody levels and neutralizing activities significantly waned over 6 months. Next, binding antibody levels were compared between ChAD- and BNT-primed vaccinated groups at 3, 4, and 6 months after the 2^nd^ dose. As shown in **Figure 2**, the ChAd-BNT cohort exhibited significantly higher total antibody levels by Roche assays than the BNT-BNT cohort, whereas IgG antibody level by Abbott assay was significantly higher in the BNT-BNT cohort. Significant differences in binding antibody levels between the two cohorts tended to narrow over time (**Figures 2A, B**). Regarding the neutralizing antibody levels, a significant difference between the two cohorts was observed only 4 months after the 2^nd^ dose (**Figure 2C**).

When we assessed changes in humoral responses after the 3^rd^ dose, binding antibody titers were markedly elevated in both groups (ChAd-BNT, 15.4 to-17.8-fold; BNT-BNT, 22.2 to 24.6-fold), and the neutralizing capacity was increased 1.3-fold in both cohorts (**Figure 2**, **Supplementary Table 1**). Binding antibody titers, which increased after the 3^rd^ booster, decreased in both cohorts over 6 months, and the ChAd-BNT cohort showed significantly lower binding antibody titers at all follow-up points compared to the BNT-BNT cohort on both the Roche and Abbott assays (*P*<0.05) (**Figures 2A, B**). The difference in geometric mean titer between the cohorts widened over the period, indicating faster declines and lower concentrations in the ChAd-BNT cohort. The neutralization activity also declined over 6 months in both cohorts, and the potency measured at 6 months after the 3^rd^ dose was significantly lower in the ChAd-BNT cohort compared to the BNT-BNT cohort (**Figure 2C**).

Comparing the results at 6 months after the 3^rd^ booster with the results at 6 months after the 2^nd^ booster, the antibody levels after the 3^rd^ dose were higher than those after the 2^nd^ dose (total antibody, 2.4 and 5.3-fold; IgG antibody, 2.5 and 4.1-fold; neutralization, 1.2 and 1.3-fold increases in the ChAd-BNT and BNT-BNT cohorts, respectively) (**Supplementary Table 2**). The results of the 3 antibody assays (Roche assay for total binding antibody, Abbott assay for IgG binding antibody, and sVNT for neutralizing antibody) were highly correlated with each other (**Supplementary Figure 1**).

### 3.2 Cellular response in infection-naïve participants

**Figure 3:**
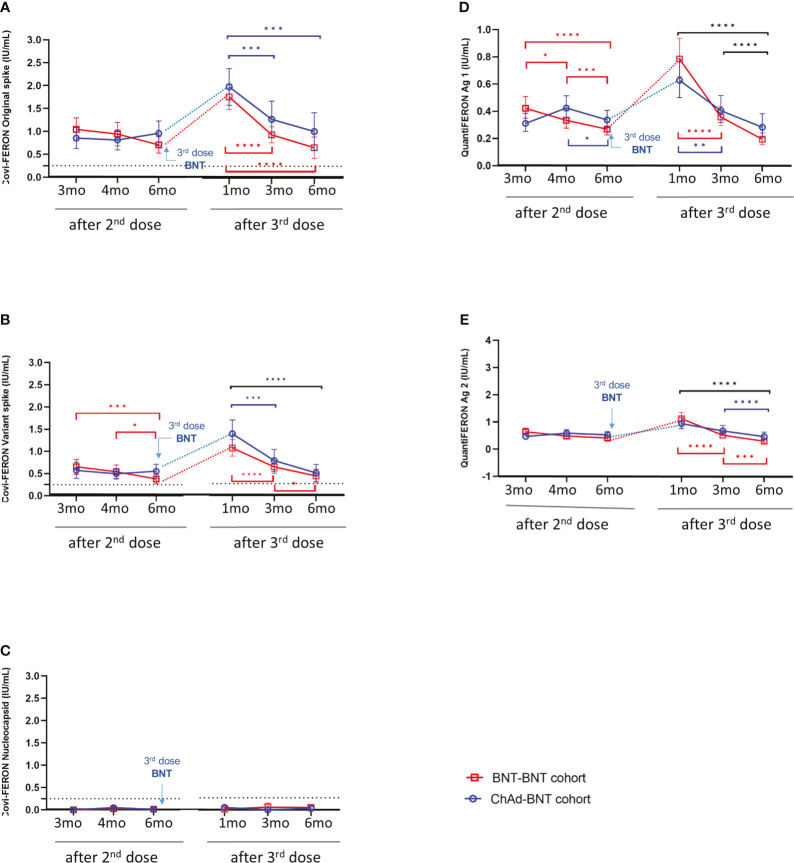
Changes in cellular immune responses in infection-naïve participants. SARS-CoV-2-specific T cell responses were measured by ELISA targeting various antigens produced by two different manufacturers **(A)** Covi-FERON stimulating original spike protein, **(B)** Covi-FERON variant spike protein, **(C)** Covi-FERON nucleocapsid protein, **(D)** QuantiFERON antigen 1 and **(E)** QuantiFERON antigen 2). Results from Covi-FERON are presented as mean with 95% confidence interval, and data measured by QuantiFERON are presented as geometric mean with a 95% confidence interval. The statistical significance was calculated using repeated measure two-way ANOVA with Sidak’s multiple comparisons test. When the ChAd-BNT cohort and BNT-BNT cohort revealed significant differences between groups, the significance was expressed either over the blue circle or over the red square. When significant change was observed over a certain period, it was expressed using ticked lines (blue: Chad-BNT cohort, red: BNT-BNT cohort, and black: both cohorts). The assay cut-off is presented as a horizontal dotted line. *P<0.05; **P<0.01; ***P<0.001; ****P <0.0001.

Changes in cellular immune responses measured by two SARS-CoV-2-specific IGRA kits are shown in **Figure 3**. After the 2^nd^ dose, persistent cellular responses were observed in the ChAd-BNT cohort. However, the BNT-BNT cohort showed a significant decrease over time in Covi-FERON (V-Sp) and QuantiFERON (Ag1) (**Figures 3A–E**). Regarding the Covi-FERON assay measuring nucleocapsid protein, infection-naïve participants showed negative results at all timepoints (**Figure 3C**).

Following the 3^rd^ dose of vaccination, SARS-CoV-2-specific cellular responses to the spike antigen [Covi-FERON (O-Sp), Covi-FERON (V-Sp), QuantiFERON (Ag1) and QuantiFERON (Ag2)] showed 1.7 to 3.0-fold increases in both ChAd-BNT and BNT-BNT cohorts. As expected, Covi-FERON nucleocapsid results were consistently negative in infection-naïve participants (**Supplementary Table 1**). The elicited cellular response targeting the spike antigen significantly decreased over time in both cohorts (**Figures 3A–E**). When comparing the two cohorts, Covi-FERON results tended to be higher in the ChAd cohort than in the BNT cohort for 3-6 months after the 3^rd^ dose. However, each SARS-CoV-2-specific IGRA result exhibited no significant difference between the two cohorts. The values of IGRA at 6 months after the 3^rd^ dose were similar to those at 6 months after the 2^nd^ dose (**Supplementary Table 2**).

Next, we analyzed the correlation between cellular immunity results measured from four SARS-CoV-2 spike antigen-specific IGRAs. A strong correlation (*r* = 0.762 – 0.898) was demonstrated between results from the same manufacturer, aside from the Covi-FERON nucleocapsid assay. The Covi-FERON results to original spike showed a strong correlation with Covi-FERON results to variant spike (*r* = 0.762 – 0.893). A lower degree of correlation was observed between Covi-FERON and QuantiFERON assays, showing *r* = 0.333 – 0.739 (**Supplementary Figure 1**).

### 3.3 Humoral and cellular immune responses in participants with breakthrough infection

In this prospective cohort, 188 (85.1%) individuals (96 ChAd-BNT and 91 BNT-BNT individuals) participated up to 6 months after the 3^rd^ dose. Of them, the frequency of SARS-CoV-2 breakthrough infection confirmed either by RT-PCR or by antibody binding to SARS-CoV-2 nucleocapsid was higher in the ChAd-BNT group than the BNT-BNT group [44.8% (43/96) vs. 28.1% (26/91), *P* = 0.0219]. Breakthrough infections occurred most frequently when the omicron variant prevailed in the study area, between 3-6 months after the 3^rd^ dose (**Table 1**).

Sequential changes in humoral and cellular immune responses before and after breakthrough infection were evaluated. The total binding antibodies and IgG antibodies were significantly increased after infection, with a median 2.3- to 2.9-fold change (**Supplementary Figure 2**). Breakthrough infection also induced an increase in neutralization activity in the ChAd-BNT cohort. The BNT-BNT cohort showed consistently high neutralizing activity before and after breakthrough infection.

Regarding cellular immune responses, Covi-FERON (NC) results were increased after breakthrough infection in both ChAd-BNT and BNT-BNT cohorts (**Supplementary Figure 3**). In terms of cellular immune response to spike protein, the ChAd-BNT cohort displayed a significantly increased response after breakthrough infection in Covi-FERON (O-SP, V-Sp) and QuantiFERON (Ag1) assays. Spike protein-specific cellular immune responses in the BNT-BNT cohort also showed an increasing tendency after breakthrough infection, but none were statistically significant.

**Figure 4:**
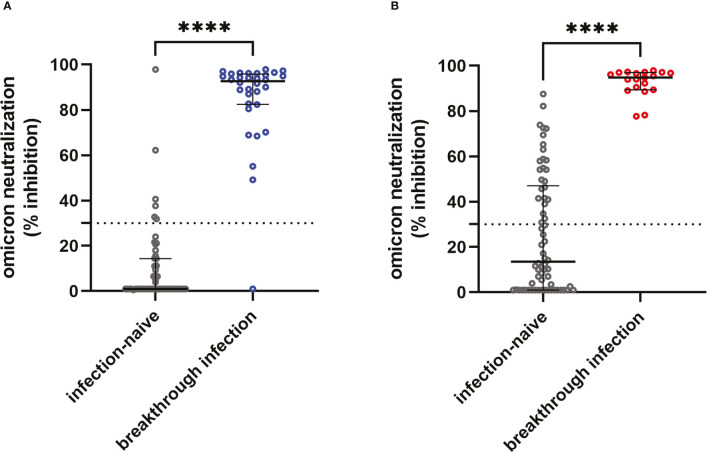
Comparison of omicron neutralization activity between infection-naïve and breakthrough infection groups. Plasma neutralization activity against omicron in samples collected in a 6^th^ sampling window. The dots represent individual participants (blue: participants with breakthrough infection in the ChAd-BNT cohort, red: participants with breakthrough infection in the BNT-BNT cohort, and black: infection-naïve participants in both cohorts). The neutralizing potency was compared between infection-naïve and breakthrough infection groups in ChAd-BNT **(A)** and BNT-BNT **(B)** cohorts. The assay cut-off is presented as a horizontal dotted line. ****P <0.0001 by Mann-Whitney U test.

Using the specimens collected 6 months after the 3^rd^ dose, the neutralization activities against omicron variants were compared between cohorts. Omicron-neutralizing activity between infection-naïve participants and participants with breakthrough infection are shown in **Figure 4**. The participants with breakthrough infection revealed significantly higher neutralization activity against omicron in both groups. Most infected participants had omicron-neutralizing activity above a 30% cut-off [41/43 (95.3%) and 26/26 (100%) in the ChAd-BNT and BNT-BNT cohorts, respectively]. On the contrary, neutralizing activity was positive only in 11.3% (6/53) and 38.5% (25/65) of ChAd-BNT and BNT-BNT cohorts of infection-naïve participants, respectively. The ChAd-BNT group had lower omicron neutralization positivity than the BNT-BNT group (*P* = 0.001).

Next, we evaluated whether the immune response 1 month after 3^rd^ dose predicted breakthrough infection up to 6 months after 3^rd^ dose. Using samples taken 1 month after the 3rd dose, immune responses were compared between infection-free and breakthrough infected participants up to 6 months after the 3rd dose. In both ChAd-primed and BNT-primed groups, there was no difference of cellular and humoral responses between infection-naïve and breakthrough infection (**Supplementary Figures 4**-**5**).

## 4 Discussion

Little is known about the difference in immune response between ChAd-primed and BNT-primed individuals following two consecutive doses of BNT. Here, we compared two groups in terms of humoral and cellular immune response and the incidence of breakthrough infection up to 6 months after the 3^rd^ BNT vaccination.

When comparing the humoral responses after the 2^nd^ dose of BNT vaccination, the total binding antibody titers were significantly higher in the ChAd-BNT cohort, while the IgG antibody titer was significantly higher in the BNT-BNT cohort. This contrasting immune response pattern has not been described in previous reports and may have resulted from different delivery systems between the two vaccine platforms. In this study, the clinical significance of this discrepancy could not be evaluated because no participants had experienced breakthrough infection prior to the 3^rd^ vaccination.

Notable differences in humoral immunity between the two cohorts were observed after the 3^rd^ vaccination. After a rapid initial increase following the 3^rd^ vaccination, waning of humoral immunity was observed in both ChAd-primed and BNT-primed groups, but ChAd-primed individuals revealed a faster decrease in both binding and neutralizing antibody levels. When the infection-naïve participants were evaluated at 6 months after the 3^rd^ dose by sVNT against omicron, most participants revealed a lack of inhibition potency due to the highly immune-evasive nature of omicron. This lack of omicron neutralization activity was more pronounced in the ChAd-primed group. The decreased humoral immune response in the ChAd-BNT group may contribute to the more frequent breakthrough infection in the group compared to the BNT-BNT group (44.8% vs. 28.1%) in the present study. This finding supports a previous report that waning humoral immunity is associated with increased breakthrough infection (16), and substantial neutralizing potency was observed only in those who acquired so-called hybrid immunity (17).

In contrast to humoral immune responses, cellular immune responses were more sustainable and durable in both groups, as described in previous studies (16, 18–20). Regarding the Covi-FERON assay measuring nucleocapsid protein, infection-naïve participants showed negative results at all timepoints. As SARS-CoV-2 infection induce strong T cell response for more than one year after infection and T cell responses to nucleocapsid antigens up to 13 months have been reported (21), Covi-FERON nucleocapsid-negative results in our study confirm infection-naïve in participants. After the 3^rd^ dose, cellular immunity levels decreased slightly and returned to similar levels as before the boost. Cellular immune responses measured by IGRA stimulated by five different SARS-CoV-2-specific antigens revealed no significant differences between ChAd-BNT and BNT-BNT groups in infection-naïve participants. In previous studies, heterologous schedules with ChAd-BNT showed higher immune responses compared to homologous ChAd-ChAd (2, 8, 22). This may be due to the low response by vector-specific immunity in ChAd vaccination. However, this study did not include participants vaccinated with the homogeneous ChAd-ChAd vaccine, and low response due to vector-specific immunity could not be confirmed. However, in the subgroup with breakthrough infection, the ChAd-BNT group showed significantly higher IFN-γ release than the BNT-BNT group. This finding supports previous reports that a stronger cellular immune response was induced by a heterogeneous primary schedule containing ChAd (23, 24).. Richardson C. also reported the greatest IFN-γ release in the ChAd-BNT group compared to homologous BNT or homologous ChAd, indicating superior cellular immunity of heterologous vaccination (25). This phenomenon may be related to an adenovirus vector-based vaccine, as Ad26.COV2.S produced by Johnson-Janssen also elicited a durable and strong CD8 T-cell response (20, 26). Weak correlations between humoral immune and cellular immune activity after vaccination have been previously reported (14, 15), probably due to different kinetics following vaccination. Cellular immunity plays a crucial role in regulating viral replication and protecting from disease progression (27–29). In this light, focusing only on humoral immunity is insufficient to evaluate vaccine effectiveness against severe COVID-19, as spike-specific T-cells are most strongly induced by heterologous vaccination (23). Considering that humoral immunity is associated with protection from COVID-19, and that cellular immunity plays a role in preventing severe disease progression in patients with breakthrough infection, the results of the ChAd-BNT group in our study are interesting. The ChAd-BNT group had a lower humoral immune response than the BNT-BNT group before infection. However, comparing the cellular responses before and after breakthrough infection, only the ChAd-BNT-BNT heterologous group showed a significant increase after infection. These results suggest that although individuals vaccinated with heterologous ChAd-BNT-BNT may be more susceptible to breakthrough infection, they elicit a stronger cellular response than the BNT-BNT-BNT group after COVID-19. Unfortunately, the role of cellular immunity in the prevention of serious disease progression could not be confirmed in this study, as none of the participants with breakthrough infection reported severe cases, including emergency room visits, hospitalizations, or death.

In terms of measuring the immune response, considered as vaccine correlates of protection (COP), we tested several commercially available kits to measure immunogenicity markers. When multiple logistic regression was performed to detect predictive markers for breakthrough infection, none of them displayed statistical significance (Data not shown). This may be due to complicated host defense mechanisms against viral disease (30), and other confounding factors may contribute to breakthrough infection. IFN-γ release stimulated by nucleocapsid protein antigens in Covi-FERON (NC) tubes was negative in all infection-naïve participants. It was significantly increased after breakthrough infection in both ChAd-BNT and BNT-BNT cohorts. This signifies that the cellular immune response against nucleocapsid protein can be indicative of natural exposure because the SARS-CoV-2 vaccines do not contain nucleocapsid protein (31). Additionally, we analyzed correlations between eight commercial kits used in this study. Three antibody assays (Roche assay for total binding antibody, Abbott assay for IgG binding antibody, and sVNT for neutralizing antibody) were highly correlated with each other, as described in previous studies (13, 32, 33). When we compared cellular responses by different assays, weak correlation was observed between Covi-FERON and QuantiFERON assays. This discrepancy could be due to different nature of peptide pools. The Covi-FERON assay predominantly measures CD4 T cell responses as the antigen goes through class II processing, whereas the QuantiFERON assay measures IFN-gamma on both CD4 and CD8 T cells from participant with HLA type binding to the peptide pool. Because cellular immune responses vary by manufacturer (e.g., Covi-FERON versus QantiFERON), careful interpretation of cellular immunogenicity and further comparative studies are required.

This study has several limitations. First, this study involved a relatively small sample size and lack of a sample of patients with severe breakout infection or more than 6 months after the 3rd dose. Second, potential bias exists in the study population as all participants were male healthy volunteers aged 20–50 years working for military service and not all participants provided longitudinal serology data. Since previous studies reported differences in antibody titers by age and gender (34, 35), future long-term studies involving disease severity in infected patients with more detailed cohorts are needed. The demographic heterogeneity of the ChAd-BNT and BNT-BNT cohorts and the possibility of including participants with previous asymptomatic infection also should be considered. In addition, neutralizing antibodies to new variants including Omicron variant were not achieved in all samples collected.

Despite these limitations, our study revealed different immune responses in the ChAd-BNT-BNT heterologous vaccination cohort compared to a cohort vaccinated with three doses of homologous BNT. A lower antibody levels in humoral immune response in the ChAd-BNT cohort following the 3^rd^ vaccination might be related to more frequent breakthrough infections. Nonetheless, the sustained cellular immune response might have prevented severe disease in both groups. Further studies are needed on the benefits of enhanced cellular immunity in the ChAd cohort. Our study provides information on booster vaccine strategies and their effectiveness, along with humoral and cellular immunity measures that may contribute to disease control.

## Data availability statement

The raw data supporting the conclusions of this article will be made available by the authors, without undue reservation.

## Ethics statement

The studies involving human participants were reviewed and approved by Institutional Review Board of the Armed Forces Medical Command (AFMC-202202-BR-012-01). The patients/participants provided their written informed consent to participate in this study.

## Author contributions

WS and HK analyzed the data and wrote the paper. HK conducted data visualization. WS, JC, KP, and JJ collected data. JL, GK, and H-SP performed the experiments. E-JO reviewed and edited the paper. E-JO and WS conceptualized the study. All authors contributed to the article and approved the submitted version.

## Supplementary material

Supplementary Figure 1 Correlograms showing the relationships between commercially available tests measuring humoral and cellular immunogenicity. The Spearman rank correlation coefficient was estimated to quantify the association between each commercialized test kit measuring immunogenicity variables and was color-coded accordingly. The Spearman correlation test was performed separately for each sampling window **(A)** the first sampling window 3 months posterior to the 2^nd^ dose of BNT vaccination, **(B)** the second sampling window 4 months posterior to the 2^nd^ dose of BNT vaccination, **(C)** the third sampling window 6 months posterior to the 2^nd^ dose of BNT vaccination, **(D)** the fourth sampling window 1 month after the 3^rd^ dose of BNT vaccination, **(E)** the 5^th^ sampling window 3 months before the 3^rd^ dose of BNT vaccination, and **(F)** the 6^th^ sampling window 6 months after the 3^rd^ dose of BNT vaccination).

Supplementary Figure 2 Changes in humoral immune response following breakthrough infection. The dots represent individual participants (blue: ChAd-BNT cohort and red: BNT-BNT cohort), and the clinical significance was calculated using the Wilcoxon signed-rank test. Each immunogenicity measurement was separately analyzed in the ChAd-BNT and BNT-BNT cohorts **(A)** total binding antibody in ChAd-BNT, **(B)** total binding antibody in BNT-BNT, **(C)** IgG binding antibody in ChAd-BNT, **(D)** IgG binding antibody in BNT-BNT, **(E)** % inhibition by sVNT in ChAd-BNT, and **(F)** % inhibition by sVNT in BNT-BNT). The assay cut-off is presented as a dotted line. ns, not significant; * *P*<0.05; ** *P*<0.01; *** *P*<0.001; **** *P <*0.0001.

Supplementary Figure 3 Changes in cellular immune response following breakthrough infection. The dots represent individual participants (blue: ChAd-BNT cohort and red: BNT-BNT cohort), and clinical significance was calculated using the Wilcoxon signed-rank test. Each immunogenicity measurement was separately analyzed in ChAd-BNT and BNT-BNT cohorts **(A)** Covi-FERON original spike protein in ChAd-BNT, **(B)** Covi-FERON original spike protein in BNT-BNT, **(C)** Covi-FERON variant spike protein in ChAd-BNT, **(D)** Covi-FERON variant spike protein in BNT-BNT, **(E)** Covi-FERON nucleocapsid protein in ChAd-BNT, **(F)** Covi-FERON nucleocapsid protein in BNT-BNT, **(G)** QuantiFERON antigen 1 in ChAd-BNT, **(H)** QuantiFERON antigen 1 in BNT-BNT, I: QuantiFERON antigen 2 in ChAd-BNT, and **(J)** QuantiFERON antigen 2 in BNT-BNT). The assay cut-off is presented as a dotted line. ns, not significant; * *P*<0.05; ** *P*<0.01; *** *P*<0.001; **** *P <*0.0001.

Supplementary Figure 4 Comparison of humoral immune response 1 month after 3^rd^ dose between infection-free (black) and breakthrough infected participant (blue: participants in the ChAd-BNT cohort, red: participants in the BNT-BNT cohort) up to 6 months after 3^rd^ dose. The dots represent individual participants, and the results were compared with Mann-Whitney U test. Each immunogenicity measurement was separately analyzed in the ChAd-BNT and BNT-BNT cohorts **(A)** total binding antibody in ChAd-BNT, **(B)** total binding antibody in BNT-BNT, **(C)** IgG binding antibody in ChAd-BNT, **(D)** IgG binding antibody in BNT-BNT, **(E)** % inhibition by sVNT in ChAd-BNT, and **(F)** % inhibition by sVNT in BNT-BNT). ns, not significant.

Supplementary Figure 5 Comparison of humoral immune response 1 month after 3^rd^ dose between infection-free (black) and breakthrough infected participant (blue: participants in the ChAd-BNT cohort, red: participants in the BNT-BNT cohort) up to 6 months after 3^rd^ dose. The dots represent individual participants, and the results were compared with Mann-Whitney U test. Each immunogenicity measurement was separately analyzed in the ChAd-BNT and BNT-BNT **(A)** Covi-FERON original spike protein in ChAd-BNT, **(B)** Covi-FERON original spike protein in BNT-BNT, **(C)** Covi-FERON variant spike protein in ChAd-BNT, **(D)** Covi-FERON variant spike protein in BNT-BNT, **(E)** Covi-FERON nucleocapsid protein in ChAd-BNT, **(F)** Covi-FERON nucleocapsid protein in BNT-BNT, **(G)** QuantiFERON antigen 1 in ChAd-BNT, **(H)** QuantiFERON antigen 1 in BNT-BNT, **(I)** QuantiFERON antigen 2 in ChAd-BNT, and **(J)** QuantiFERON antigen 2 in BNT-BNT). The assay cut-off is presented as a dotted line. ns, not significant.
