# Supplementary figures and images for: Comparison of humoral and cellular immune responses between ChAd-BNT heterologous vaccination and BNT-BNT homologous vaccination following the third BNT dose: A prospective cohort study
Source: Front Immunol. 2023 Mar 2;14:1120556. doi: 10.3389/fimmu.2023.1120556 (PMC10017529; doi:10.3389/fimmu.2023.1120556)

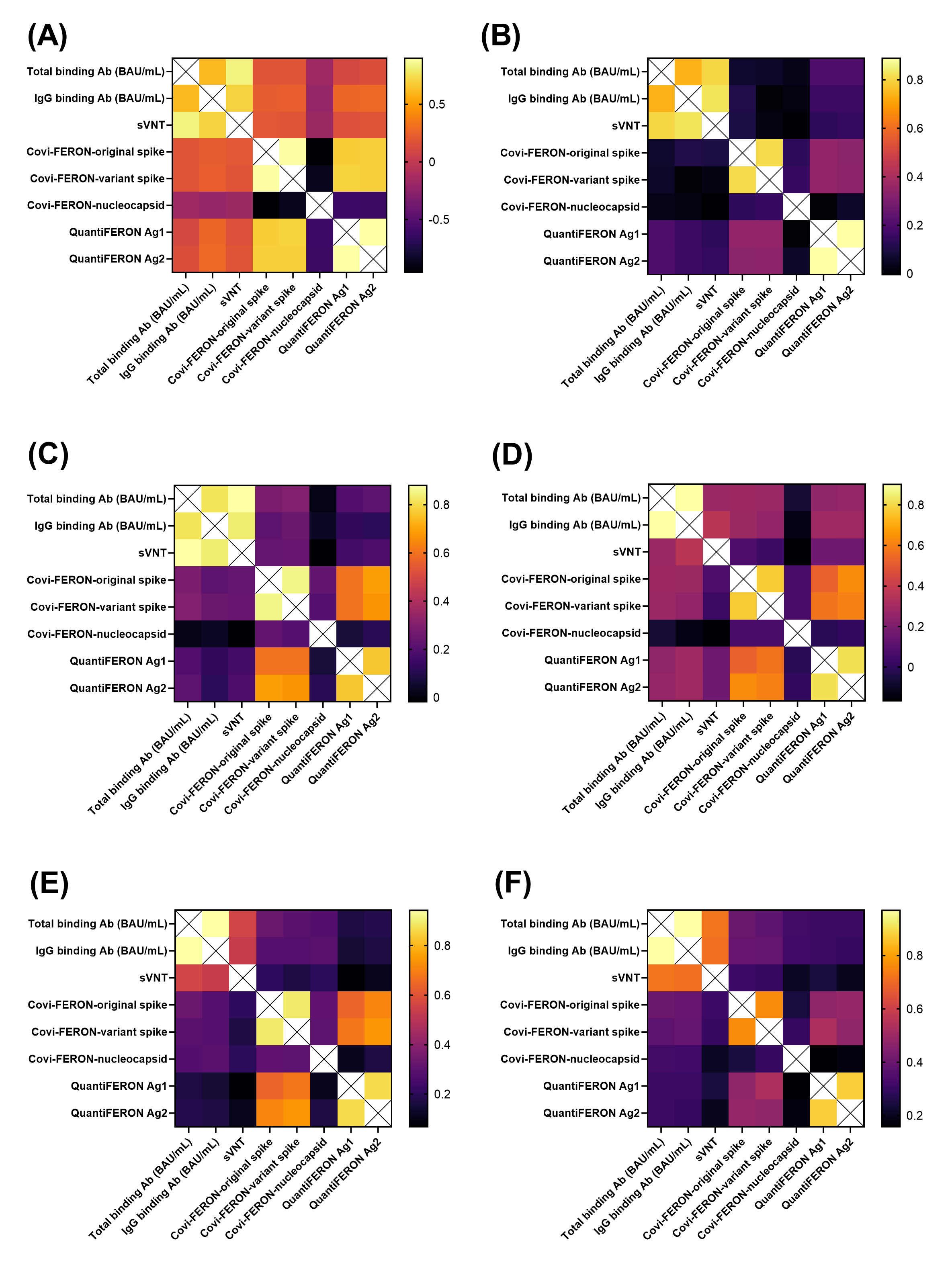

Supplement: Supplementary Figure 1 — Correlograms showing the relationships between commercially available tests measuring humoral and cellular immunogenicity. The Spearman rank correlation coefficient was estimated to quantify the association between each commercialized test kit measuring immunogenicity variables and was color-coded accordingly. The Spearman correlation test was performed separately for each sampling window (A) the first sampling window 3 months posterior to the 2nd dose of BNT vaccination, (B) the second sampling window 4 months posterior to the 2nd dose of BNT vaccination, (C) the third sampling window 6 months posterior to the 2nd dose of BNT vaccination, (D) the fourth sampling window 1 month after the 3rd dose of BNT vaccination, (E) the 5th sampling window 3 months before the 3rd dose of BNT vaccination, and (F) the 6th sampling window 6 months after the 3rd dose of BNT vaccination). [file Image_1.tif]

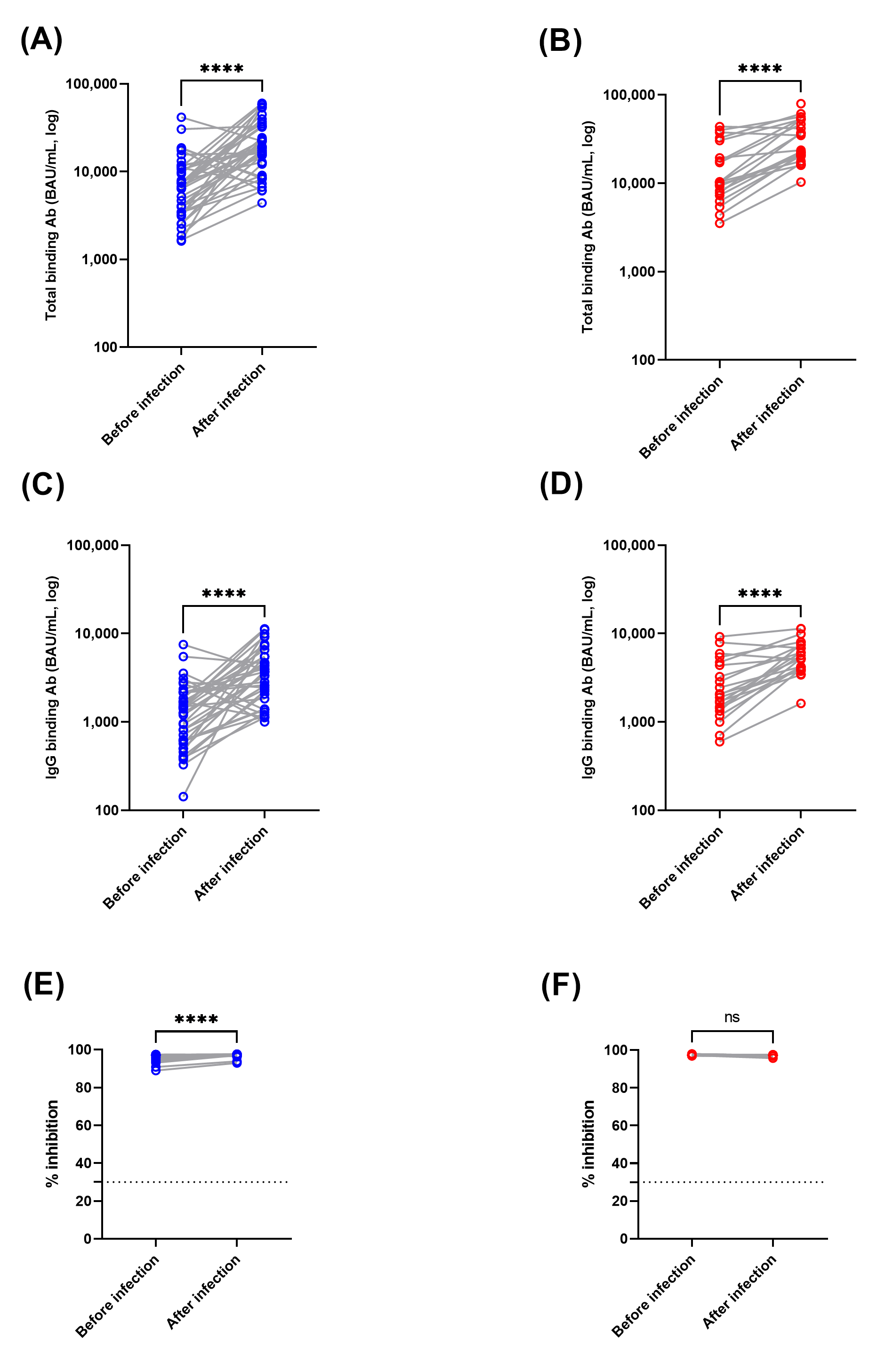

Supplement: Supplementary Figure 2 — Changes in humoral immune response following breakthrough infection. The dots represent individual participants (blue: ChAd-BNT cohort and red: BNT-BNT cohort), and the clinical significance was calculated using the Wilcoxon signed-rank test. Each immunogenicity measurement was separately analyzed in the ChAd-BNT and BNT-BNT cohorts (A) total binding antibody in ChAd-BNT, (B) total binding antibody in BNT-BNT, (C) IgG binding antibody in ChAd-BNT, (D) IgG binding antibody in BNT-BNT, (E) % inhibition by sVNT in ChAd-BNT, and (F) % inhibition by sVNT in BNT-BNT). The assay cut-off is presented as a dotted line. ns, not significant; * P<0.05; ** P<0.01; *** P<0.001; **** P <0.0001. [file Image_2.tif]

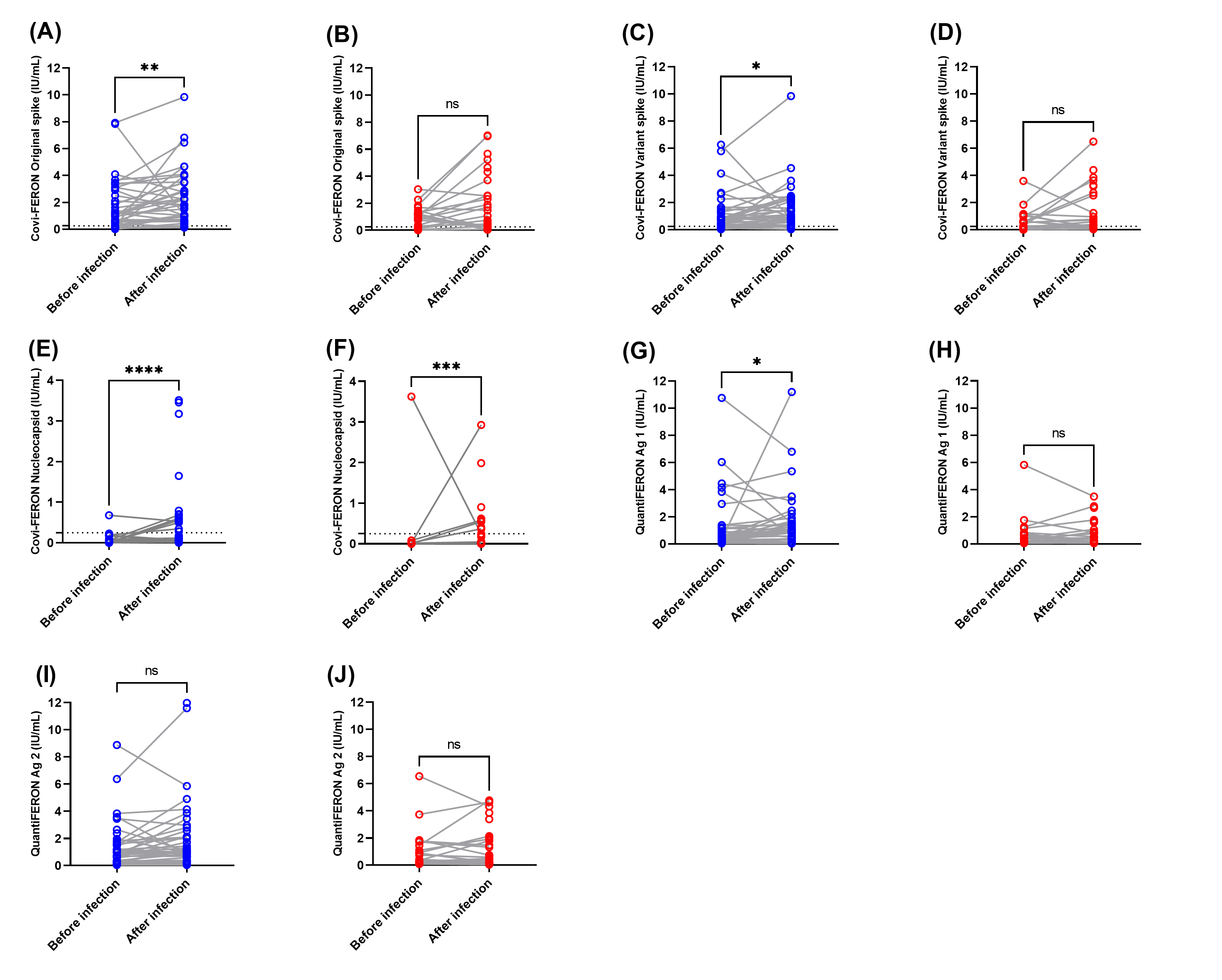

Supplement: Supplementary Figure 3 — Changes in cellular immune response following breakthrough infection. The dots represent individual participants (blue: ChAd-BNT cohort and red: BNT-BNT cohort), and clinical significance was calculated using the Wilcoxon signed-rank test. Each immunogenicity measurement was separately analyzed in ChAd-BNT and BNT-BNT cohorts (A) Covi-FERON original spike protein in ChAd-BNT, (B) Covi-FERON original spike protein in BNT-BNT, (C) Covi-FERON variant spike protein in ChAd-BNT, (D) Covi-FERON variant spike protein in BNT-BNT, (E) Covi-FERON nucleocapsid protein in ChAd-BNT, (F) Covi-FERON nucleocapsid protein in BNT-BNT, (G) QuantiFERON antigen 1 in ChAd-BNT, (H) QuantiFERON antigen 1 in BNT-BNT, I: QuantiFERON antigen 2 in ChAd-BNT, and (J) QuantiFERON antigen 2 in BNT-BNT). The assay cut-off is presented as a dotted line. ns, not significant; * P<0.05; ** P<0.01; *** P<0.001; **** P <0.0001. [file Image_3.tif]

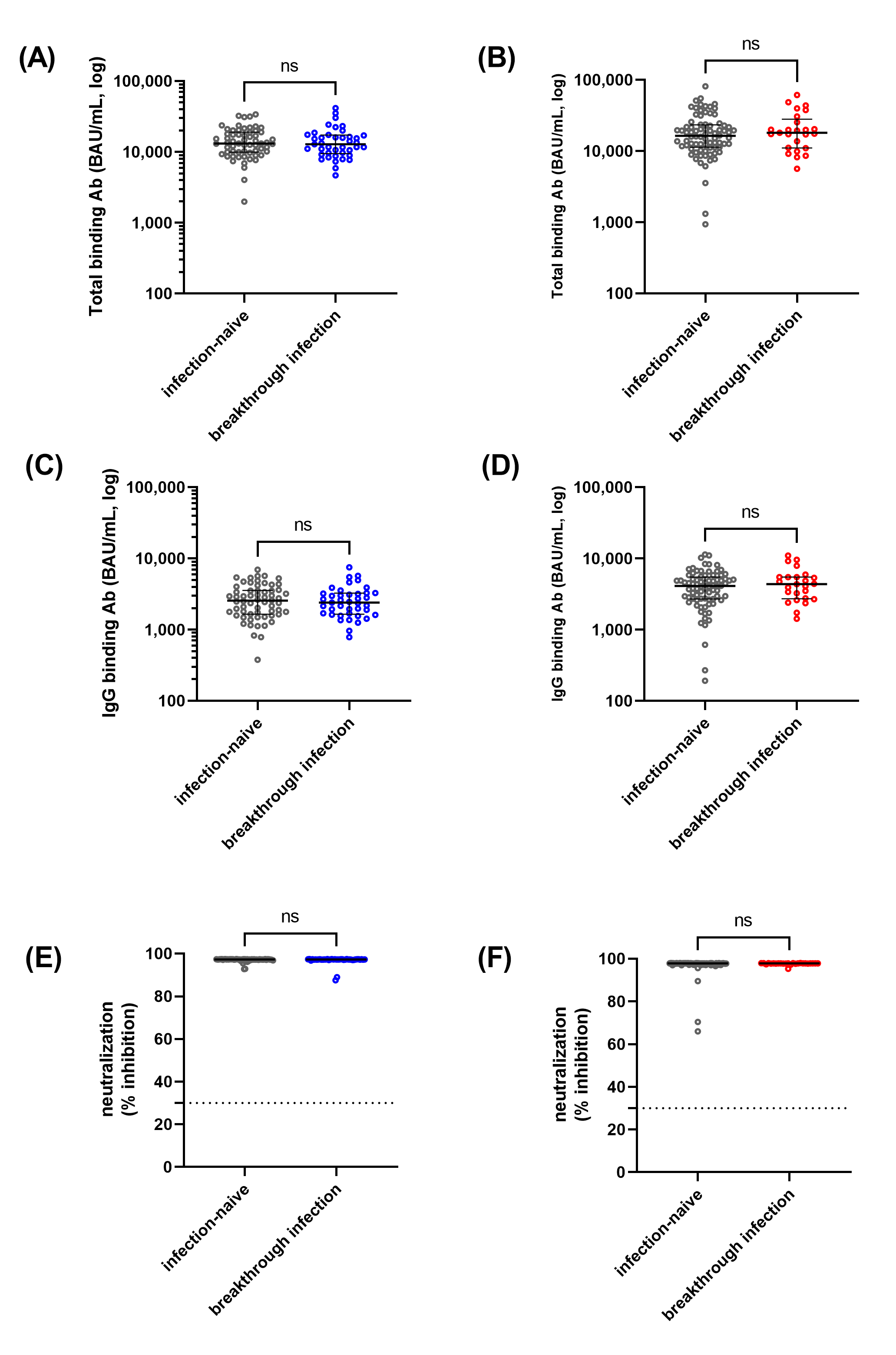

Supplement: Supplementary Figure 4 — Comparison of humoral immune response 1 month after 3rd dose between infection-free (black) and breakthrough infected participant (blue: participants in the ChAd-BNT cohort, red: participants in the BNT-BNT cohort) up to 6 months after 3rd dose. The dots represent individual participants, and the results were compared with Mann-Whitney U test. Each immunogenicity measurement was separately analyzed in the ChAd-BNT and BNT-BNT cohorts (A) total binding antibody in ChAd-BNT, (B) total binding antibody in BNT-BNT, (C) IgG binding antibody in ChAd-BNT, (D) IgG binding antibody in BNT-BNT, (E) % inhibition by sVNT in ChAd-BNT, and (F) % inhibition by sVNT in BNT-BNT). ns, not significant. [file Image_4.tif]

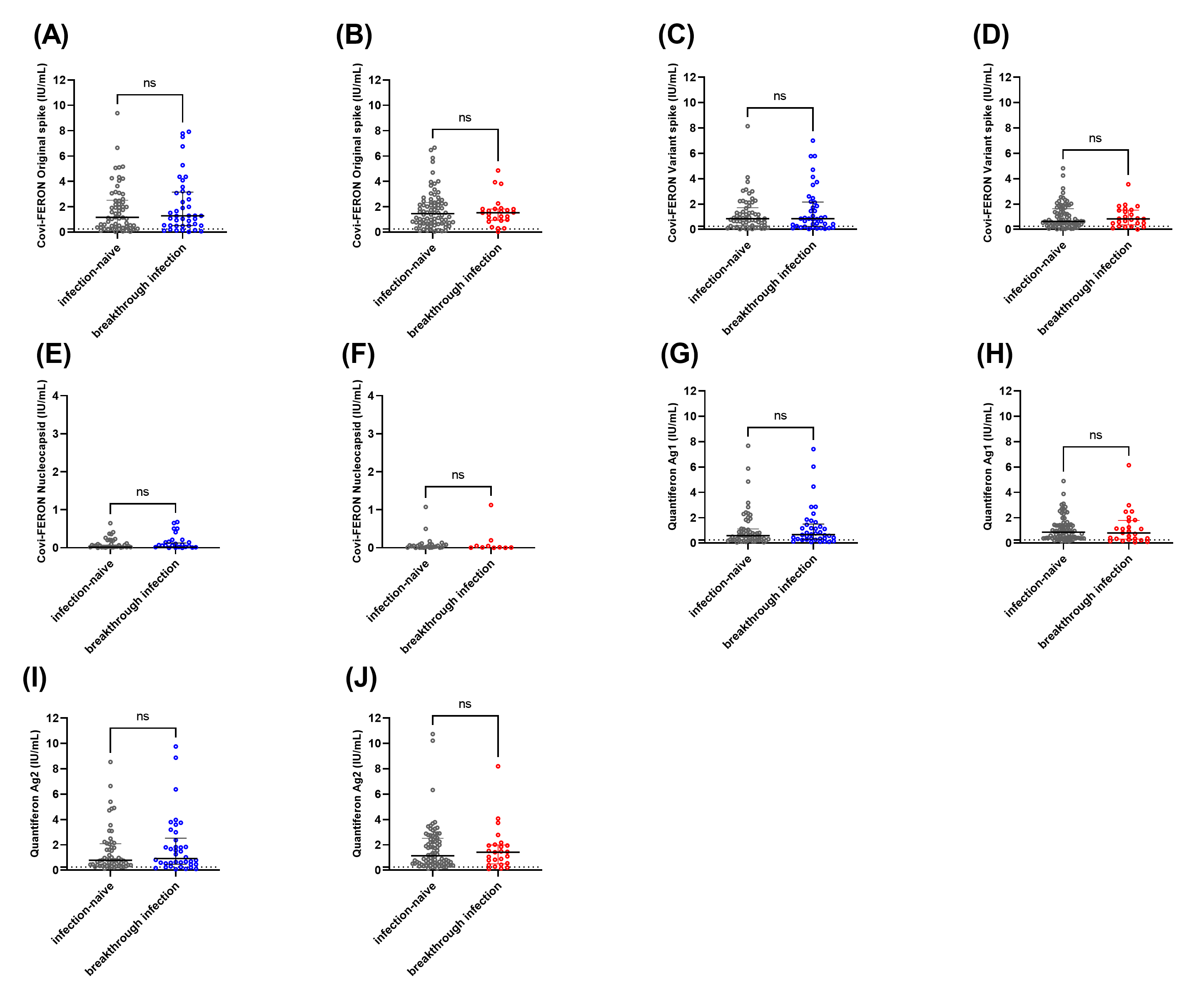

Supplement: Supplementary Figure 5 — Comparison of humoral immune response 1 month after 3rd dose between infection-free (black) and breakthrough infected participant (blue: participants in the ChAd-BNT cohort, red: participants in the BNT-BNT cohort) up to 6 months after 3rd dose. The dots represent individual participants, and the results were compared with Mann-Whitney U test. Each immunogenicity measurement was separately analyzed in the ChAd-BNT and BNT-BNT (A) Covi-FERON original spike protein in ChAd-BNT, (B) Covi-FERON original spike protein in BNT-BNT, (C) Covi-FERON variant spike protein in ChAd-BNT, (D) Covi-FERON variant spike protein in BNT-BNT, (E) Covi-FERON nucleocapsid protein in ChAd-BNT, (F) Covi-FERON nucleocapsid protein in BNT-BNT, (G) QuantiFERON antigen 1 in ChAd-BNT, (H) QuantiFERON antigen 1 in BNT-BNT, (I) QuantiFERON antigen 2 in ChAd-BNT, and (J) QuantiFERON antigen 2 in BNT-BNT). The assay cut-off is presented as a dotted line. ns, not significant. [file Image_5.tif]
