# Supplementary material for: Comparison of humoral and cellular immune responses between ChAd-BNT heterologous vaccination and BNT-BNT homologous vaccination following the third BNT dose: A prospective cohort study
Source: Front Immunol. 2023 Mar 2;14:1120556. doi: 10.3389/fimmu.2023.1120556 (PMC10017529; doi:10.3389/fimmu.2023.1120556)
Supplement: Supplementary file 6 [file DataSheet_1.docx]

Supplementary Material

Comparison of humoral and cellular immune responses between ChAd-BNT heterologous vaccination and BNT-BNT homologous vaccination following the third BNT dose: A prospective cohort study

**Supplementary Figure 1.** Correlograms showing the relationships between commercially available tests measuring humoral and cellular immunogenicity.

The Spearman rank correlation coefficient was estimated to quantify the association between each commercialized test kit measuring immunogenicity variables and was color-coded accordingly. The Spearman correlation test was performed separately for each sampling window (A: the first sampling window 3 months posterior to the 2^nd^ dose of BNT vaccination, B: the second sampling window 4 months posterior to the 2^nd^ dose of BNT vaccination, C: the third sampling window 6 months posterior to the 2^nd^ dose of BNT vaccination, D: the fourth sampling window 1 month after the 3^rd^ dose of BNT vaccination, E: the 5^th^ sampling window 3 months before the 3^rd^ dose of BNT vaccination, and F: the 6^th^ sampling window 6 months after the 3^rd^ dose of BNT vaccination).

**Supplementary Figure 2.** Changes in humoral immune response following breakthrough infection.

The dots represent individual participants (blue: ChAd-BNT cohort and red: BNT-BNT cohort), and the clinical significance was calculated using the Wilcoxon signed-rank test. Each immunogenicity measurement was separately analyzed in the ChAd-BNT and BNT-BNT cohorts (A: total binding antibody in ChAd-BNT, B: total binding antibody in BNT-BNT, C: IgG binding antibody in ChAd-BNT, D: IgG binding antibody in BNT-BNT, E: % inhibition by sVNT in ChAd-BNT, and F: % inhibition by sVNT in BNT-BNT). The assay cut-off is presented as a dotted line. ns, not significant; * *P*<0.05; ** *P*<0.01; *** *P*<0.001; **** *P* <0.0001.

**Supplementary Figure 3.** Changes in cellular immune response following breakthrough infection.

The dots represent individual participants (blue: ChAd-BNT cohort and red: BNT-BNT cohort), and clinical significance was calculated using the Wilcoxon signed-rank test. Each immunogenicity measurement was separately analyzed in ChAd-BNT and BNT-BNT cohorts (A: Covi-FERON original spike protein in ChAd-BNT, B: Covi-FERON original spike protein in BNT-BNT, C: Covi-FERON variant spike protein in ChAd-BNT, D: Covi-FERON variant spike protein in BNT-BNT, E: Covi-FERON nucleocapsid protein in ChAd-BNT, F: Covi-FERON nucleocapsid protein in BNT-BNT, G: QuantiFERON antigen 1 in ChAd-BNT, H: QuantiFERON antigen 1 in BNT-BNT, I: QuantiFERON antigen 2 in ChAd-BNT, and J: QuantiFERON antigen 2 in BNT-BNT). The assay cut-off is presented as a dotted line. ns, not significant; * *P*<0.05; ** *P*<0.01; *** *P*<0.001; **** *P* <0.0001.

**Supplementary Figure 4.** Comparison of humoral immune response 1 month after 3^rd^ dose between infection-free (black) and breakthrough infected participant (blue: participants in the ChAd-BNT cohort, red: participants in the BNT-BNT cohort) up to 6 months after 3^rd^ dose.

The dots represent individual participants, and the results were compared with Mann-Whitney U test. Each immunogenicity measurement was separately analyzed in the ChAd-BNT and BNT-BNT cohorts (A: total binding antibody in ChAd-BNT, B: total binding antibody in BNT-BNT, C: IgG binding antibody in ChAd-BNT, D: IgG binding antibody in BNT-BNT, E: % inhibition by sVNT in ChAd-BNT, and F: % inhibition by sVNT in BNT-BNT). ns, not significant.

**Supplementary Figure 5.** Comparison of humoral immune response 1 month after 3^rd^ dose between infection-free (black) and breakthrough infected participant (blue: participants in the ChAd-BNT cohort, red: participants in the BNT-BNT cohort) up to 6 months after 3^rd^ dose.

The dots represent individual participants, and the results were compared with Mann-Whitney U test. Each immunogenicity measurement was separately analyzed in the ChAd-BNT and BNT-BNT

(A: Covi-FERON original spike protein in ChAd-BNT, B: Covi-FERON original spike protein in BNT-BNT, C: Covi-FERON variant spike protein in ChAd-BNT, D: Covi-FERON variant spike protein in BNT-BNT, E: Covi-FERON nucleocapsid protein in ChAd-BNT, F: Covi-FERON nucleocapsid protein in BNT-BNT, G: QuantiFERON antigen 1 in ChAd-BNT, H: QuantiFERON antigen 1 in BNT-BNT, I: QuantiFERON antigen 2 in ChAd-BNT, and J: QuantiFERON antigen 2 in BNT-BNT). The assay cut-off is presented as a dotted line. ns, not significant.

**Supplementary table 1. Fold increase in immunogenicity measures following 3^rd^ dose booster vaccination in** **infection-naïve participants.**

|  | 6-month posterior to 2^nd^ dose administration* | 1-month posterior to 3^rd^ dose administration* | Fold change |
| --- | --- | --- | --- |
| SARS-CoV-2 spike specific total binding antibody (BAU/mL) | | | |
| ChAd-BNT cohort | |  |  |
|  | 857.1 | 13167 | 15.4-fold increased |
| BNT-BNT cohort | |  |  |
|  | 715.3 | 17579 | 24.6-fold increased |
| SARS-CoV-2 spike specific IgG binding antibody (BAU/mL) | | | |
| ChAd-BNT cohort | |  |  |
|  | 135.9 | 2421 | 17.8-fold increased |
| BNT-BNT cohort | |  |  |
|  | 177.3 | 3934 | 22.2-fold increased |
| SARS-CoV-2 neutralization (% inhibition) | | | |
| ChAd-BNT cohort | |  |  |
|  | 75.2 | 96.9 | 1.3-fold increased |
| BNT-BNT cohort | |  |  |
|  | 75.3 | 97.3 | 1.3-fold increased |
| T-cell reactivity against covi-FERON original spike protein (IU/mL) | | | |
| ChAd-BNT cohort | |  |  |
|  | 0.96 | 1.93 | 2.0-fold increased |
| BNT-BNT cohort | |  |  |
|  | 0.70 | 1.75 | 2.5-fold increased |
| T-cell reactivity against covi-FERON variant spike protein (IU/mL) | | | |
| ChAd-BNT cohort | |  |  |
|  | 0.55 | 1.33 | 2.4-fold increased |
| BNT-BNT cohort | |  |  |
|  | 0.38 | 1.04 | 2.7-fold increased |
| T-cell reactivity against covi-FERON nucleocapsid protein (IU/mL) | | | |
| ChAd-BNT cohort | |  |  |
|  | 0.01 | 0.05 | Not available† |
| BNT-BNT cohort | |  |  |
|  | 0.02 | 0.02 | Not available† |
| T-cell reactivity on QuantiFERON antigen 1 tube (IU/mL) | | | |
| ChAd-BNT cohort | |  |  |
|  | 0.34 | 0.62 | 1.8-fold increased |
| BNT-BNT cohort | |  |  |
|  | 0.27 | 0.80 | 3.0-fold increased |
| T-cell reactivity on QuantiFERON antigen 2 tube (IU/mL) | | | |
| ChAd-BNT cohort | |  |  |
|  | 0.53 | 0.92 | 1.7-fold increased |
| BNT-BNT cohort | |  |  |
|  | 0.41 | 1.13 | 2.8-fold increased |

* Arithmetric mean values were used for Covi-FERON assays. Otherwise, geometric mean titers were used.

† The mean of Covi-FERON nucleocapsid assay is lower than the cut-off value, thus the fold change is not meaningful in the context.

**Supplementary table 2. Fold change in immunogenicity measures at 6 months posterior to 2^nd^ dose and 3^rd^ dose booster vaccination in infection-naïve participants.**

|  | 6-month posterior to 2^nd^ dose administration* | 6-month posterior to 3^rd^ dose administration* | Fold change |
| --- | --- | --- | --- |
| SARS-CoV-2 spike specific total binding antibody (BAU/mL) | | | |
| ChAd-BNT cohort | |  |  |
|  | 857.1 | 2043 | 2.4-fold increased |
| BNT-BNT cohort | |  |  |
|  | 715.3 | 3782 | 5.3-fold increased |
| SARS-CoV-2 spike specific IgG binding antibody (BAU/mL) | | | |
| ChAd-BNT cohort | |  |  |
|  | 135.9 | 334.6 | 2.5-fold increased |
| BNT-BNT cohort | |  |  |
|  | 177.3 | 727.1 | 4.1-fold increased |
| SARS-CoV-2 neutralization (% inhibition) | | | |
| ChAd-BNT cohort | |  |  |
|  | 75.2 | 91.2 | 1.2-fold increased |
| BNT-BNT cohort | |  |  |
|  | 75.3 | 95.7 | 1.3-fold increased |
| T-cell reactivity against covi-FERON original spike protein (IU/mL) | | | |
| ChAd-BNT cohort | |  |  |
|  | 0.96 | 0.99 | 1.0-fold increased |
| BNT-BNT cohort | |  |  |
|  | 0.70 | 0.65 | 0.9-fold decreased |
| T-cell reactivity against covi-FERON variant spike protein (IU/mL) | | | |
| ChAd-BNT cohort | |  |  |
|  | 0.55 | 0.48 | 0.9-fold decreased |
| BNT-BNT cohort | |  |  |
|  | 0.38 | 0.42 | 1.1-fold increased |
| T-cell reactivity against covi-FERON nucleocapsid protein (IU/mL) | | | |
| ChAd-BNT cohort | |  |  |
|  | 0.01 | 0.03 | Not available† |
| BNT-BNT cohort | |  |  |
|  | 0.02 | 0.05 | Not available† |
| T-cell reactivity on QuantiFERON antigen 1 tube (IU/mL) | | | |
| ChAd-BNT cohort | |  |  |
|  | 0.34 | 0.28 | 0.8-fold decreased |
| BNT-BNT cohort | |  |  |
|  | 0.27 | 0.19 | 0.8-fold decreased |
| T-cell reactivity on QuantiFERON antigen 2 tube (IU/mL) | | | |
| ChAd-BNT cohort | |  |  |
|  | 0.53 | 0.45 | 0.7-fold decreased |
| BNT-BNT cohort | |  |  |
|  | 0.41 | 0.28 | 0.7-fold decreased |

* Arithmetric mean values were used for Covi-FERON assays. Otherwise, geometric mean titers were used.

† The mean of Covi-FERON nucleocapsid assay is lower than the cut-off value, thus the fold change is not meaningful in the context.
